# Graphene-Induced Room Temperature Ferromagnetism in Cobalt Nanoparticles Decorated Graphene Nanohybrid

**DOI:** 10.1186/s11671-020-03398-7

**Published:** 2020-08-17

**Authors:** Amar Nath Yadav, Ashwani Kumar Singh, Pramod Kumar, Kedar Singh

**Affiliations:** 1 School of Physical Sciences, Jawaharlal Nehru University, New Delhi, 110067 India; 2 Sri Aurobindo College, -110017, New Delhi, India

**Keywords:** Nanoparticles, Carbon materials, Ferromagnetism

## Abstract

Control over the magnetic interactions in magnetic nanoparticles (MNPs) is a crucial issue to the future development of nanometer-sized integrated “spintronic” applications. Here, we have developed a nanohybrid structure to achieve room temperature ferromagnetism, via a facile, effective, and reproducible solvothermal synthesis method. The plan has been put onto cobalt (Co) NPs, where the growth of Co NPs on the surface of reduced graphene oxide (rGO) nanosheets switches the magnetic interactions from superparamagnetic to ferromagnetic at room temperature. Switching-on ferromagnetism in this nanohybrid may be due to the hybridization between unsaturated 2p_z_ orbitals of graphene and 3d orbitals of Co, which promotes ferromagnetic long-range ordering. The ferromagnetic behavior of Co-rGO nanohybrid makes it excellent material in the field of spintronics, catalysis, and magnetic resonance imaging.

## Introduction

In the recent decade, magnetic nanoparticles (MNPs) have attracted the considerable interest of scientists because of its potential applications in spintronics, catalysis, and biology [1, 2]. In various metal NPs (Fe, Co, Ni), Co NPs have been extensively studied due to their potential industrial applications. Nowadays, it has been found that Co NPs are an excellent alternative to iron NPs because of its large anisotropy and greater proton relaxation [3]. These interesting properties of Co NPs make them an ideal candidate for applications in catalysis, magnetic resonance imaging (MRI), drug delivery, and therapeutic [4–6].

In 2D carbon materials, graphene has been found as a perfect supporting material for semiconductor and metal oxide nanoparticles because of its large surface area, light-weight, less toxicity, and hydrophilic nature [7]. The excellent and unique properties of 2D graphene come out from its tightly packed carbon atoms that form an sp^2^-hybrid network in a honeycomb lattice. In metal NPs-graphene hybrid, the NPs are attached with the surface of the graphene sheet through strong covalent bonding which further avoids evaporation and migration of NPs. Moreover, graphene has unsaturated p_z_ orbital and zero bandgaps, both of these properties are useful for the electronic interaction with 3d orbital of transition metal NPs [8]. Furthermore, the resulting hybrid material may hold unique properties of graphene such as long spin coherence lengths and times because of limited fine interactions and small spin-orbit coupling [8]. Thus, graphene is a promising material to alternate the electronic band structure of magnetic NPs efficiently and can promote the room temperature ferromagnetic interactions.

For the synthesis of MNPs-graphene nanocomposite, various methods have been explored by the researcher, including sol-gel method, electrochemical deposition, green synthesis method, in situ assembly method, and solvothermal method [9–13]. Depending on the required applications, one can select a suitable manner to synthesize MNPs-graphene nanocomposite, since the methods described above have its benefit and drawback. In a recent study, Xu et al*.* [14] have synthesized Co-rGO nanocomposite by the one-step solvothermal method and found this composite as an excellent catalyst for the reduction of Cr (VI) to Cr (III). Athinarayanan et al*.* [12] have prepared Co_3_O_4_-rGO nanocomposite using date palm fruit syrup and evaluated its biological properties on human mesenchymal stem cells.

In this work, we have designed Co-rGO nanohybrid by a simple and effective solvothermal synthesis method. In Co-rGO nanohybrid, graphene was used as a supporting material that provides ample surface area, monodispersity for Co NPs, and also prevents them from oxidation and aggregation. The detailed microstructural experimental results reveal the successful formation of Co-rGO nanohybrid. Further, the magnetic properties of Co NPs and Co-rGO nanohybrid were examined by vibrating sample magnetometer technique, where the room temperature M-H curve shows superparamagnetic behavior for Co NPs. Further, by the decoration of Co NPs on the surface of graphene, we have observed ferromagnetic behavior at room temperature.

## Methods

### Materials

Cobalt (III) acetylacetonate (99.99%, Sigma Aldrich), Oleylamine (> 50.0% (GC), TCI), ethanol (99.9%, Merc). Ethylene glycol (Fisher Scientific), sodium acetate anhydrous (98.5%, Fisher Scientific), ethylene diamine (99%, Merc), double distilled water (99%, Merc). Sulfuric acid (H_2_SO_4_, Fisher Scientific), nitric acid (HNO_3_, Fisher Scientific), hydrochloric acid (HCl, Fisher Scientific), potassium chlorate (KClO_3_, Fisher Scientific).

### Synthesis of Graphite Oxide

Graphite oxide was prepared by using Staudenmaier’s method with slight modification [13, 15, 16]. In a 500-ml beaker, 180 ml of sulfuric acid and 90 ml of nitric acid were added under an ice bath. Further, 5 g graphite powder was added to the mixture and allowed to mix by magnetic stirring. Then, 55 g potassium chlorate was added to reaction mixtures in 2 h. After that, the ice bath was removed, and the reaction mixture was allowed to string for 5 days. Finally, the solution was washed well with HCl and distilled water solution (10 times), and the obtained product was dried under vacuum furnace at 80 °C.

### Synthesis of Cobalt Nanoparticles

Synthesis of Co NPs was conducted by one-step solvothermal method [17]. Briefly, 1.8 mmol (641.26 mg) of cobalt (III) acetylacetonate was added to the 75 ml of oleylamine in a beaker. The reaction mixture was heated at 100 °C under magnetic stirring about 1 h. Further, the mixture was transferred into a 100 ml autoclave and heated at 220 °C for 20 h. Finally, the solution was purified by ethanol, and the obtained precipitate was dried in a vacuum furnace at 60 °C.

### Synthesis of Co-rGO Nanohybrid

Co-rGO nanohybrid was synthesized by a simple solvothermal synthesis method as described by our group in the previous study [13, 15, 16]. In a typical synthesis protocol, 80 ml ethylene glycol, 15 ml ethylene-diamine, 6 g sodium acetate, 200 mg graphite oxide, and 50 mg as-synthesized cobalt nanoparticles have been sonicated in a beaker for 3 h. Further, the dispersed solution was transferred into a 100 ml autoclave and heated at temperature 200 °C for 12 h. Finally, the reaction mixture was allowed to cool at ambient temperature, purified by ethanol several times, and the obtained product was dried in a vacuum furnace at 60 °C.

Rigaku MiniFlex tabletop X-ray diffractometer (XRD) with Cu Kα (*λ* = 1.54 Å) was used to obtain XRD pattern of as-synthesized powder samples. The size and shape of as-prepared samples were acquired from a JEOL-2100F electron microscope. For this characterization, the accelerating voltage was used as 120 kV, and samples were prepared by drop costing of dispersed sample on a 300-mesh carbon-coated copper grid. The surface morphologies and elemental mapping of as-synthesized samples were determined from SEM, Zeiss EVO 40 microscope where the operating voltage was 20 kV. Raman spectroscopy was carried out by a Wi-tech alpha 300 RA raman spectrometer having an argon laser of wavelength 532 nm. Magnetic properties of Co NPs, rGO, and Co-rGO composite were obtained by the vibrating sample magnetometer (VSM) technique attached with PPMS cryogenics limited, USA.

## Results and Discussion

**Fig. 1:**
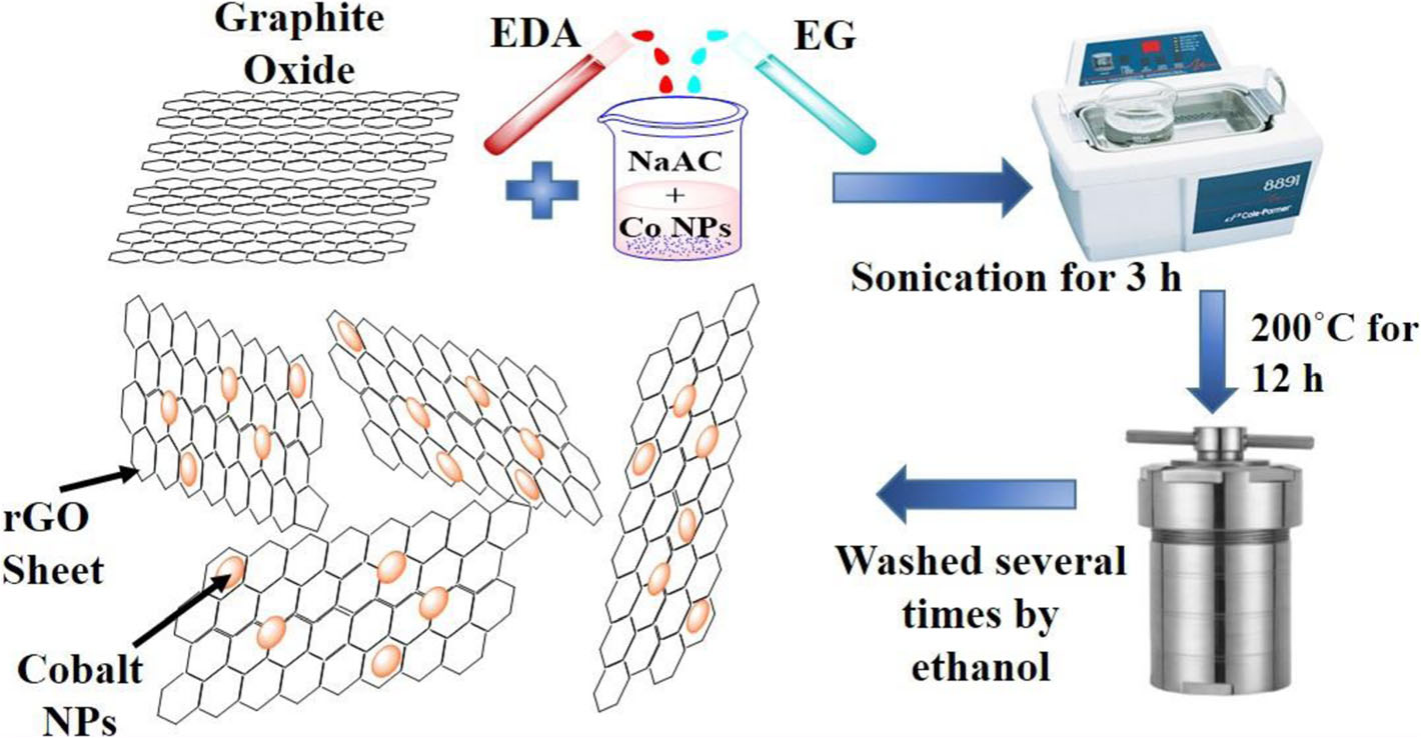
Schematic diagram illustrating synthesis mechanism of Co-rGO nanohybrid

Figure 1 illustrates the synthesis mechanism of Co-rGO nanohybrid. As depicted in the figure, first graphite oxide (GO), Co NPs, ethylenediamine (EDA), ethylene glycol (EG), and sodium acetate (NaAc) were taken in a beaker and sonicated inside a sonicator for proper dispersion of the mixture. Here, NaAc was used as an electrostatic stabilizer which can stop particle agglomeration; EDA and EG act as the solvent media for proper dispersion of Co NPs. After proper dispersion, the mixture was transferred in a furnace under 200 °C for 12 h. In this solvothermal reaction, EDA plays a significant role in the evolution of Co-rGO nanohybrid, and EG acts as a reducing agent which contributes to the reduction of GO into rGO [15, 16].

**Fig. 2:**
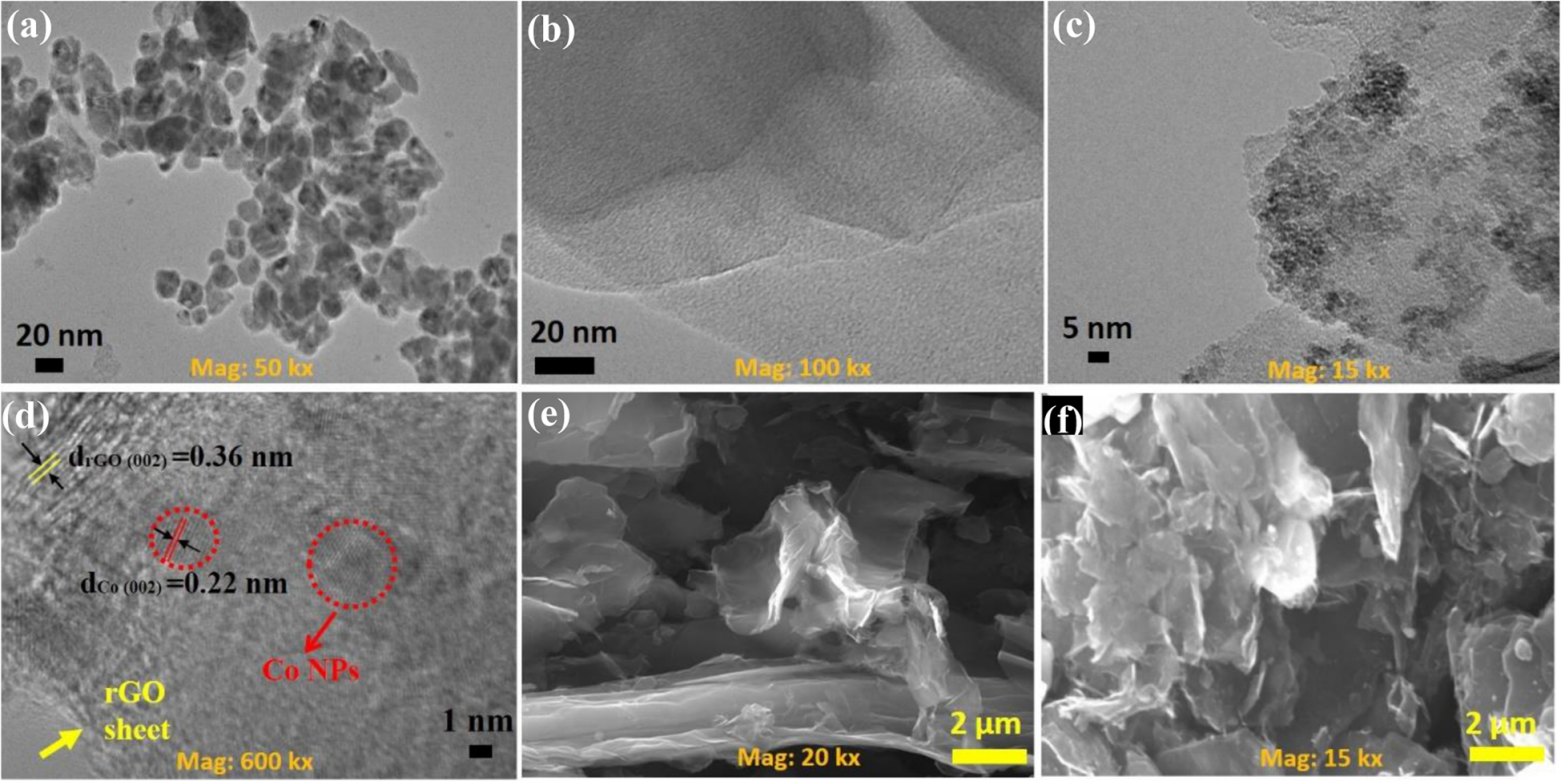
**a**–**d** TEM images **a** Co NPs, **b** rGO nanosheets, **c** and **d** Co-rGO nanohybrid. **e**– **f** SEM images **e** rGO nanosheets and **f** Co-rGO nanohybrid

The size, shape, and disparity of as-prepared Co NPs have been investigated by transmission electron microscope (TEM). It can be seen in Fig. 2a, most of the Co NPs have a nearly spherical shape with an average size of 15–20 nm. Figure 2b is the TEM image of rGO nanosheets, which shows that the rGO sheet is almost transparent with a wrinkled paper-like surface. Further, Fig. 2 c and d are respectively TEM and HRTEM images of Co-rGO nanohybrid. It is well depicted that Co NPs are successfully decorated over the surface of the rGO sheet. The average diameter of Co NPs on the surface of the rGO sheet was found to be 5–8 nm, which is lower than the as observed value in the case of Co NPs. This change is observed due to graphene and ethylene glycol, which limit the size of Co NPs in solvothermal reaction [14]. Furthermore, from the HRTEM image (Fig. 2d), the interplanar spacing was calculated as 0.36 and 0.22 nm for rGO and Co NPs, respectively, which corresponds to (002) plane of both materials. The surface morphology of as-synthesized rGO and Co-rGO nanohybrid was investigated by scanning electron microscope (SEM). Figure 2 e is a typical SEM image of rGO nanosheets. It illustrates that rGO has fluffy morphology with a lamellar structure. SEM image of Co-rGO nanohybrid is shown in Fig. 2f. The whitish patches over the rGO sheet are a clear indication of the good dispersion of Co NPs. It also indicates a strong covalent bonding between Co NPs and the rGO sheet through electronic interaction. Therefore, rGO plays a vital role in Co-rGO nanohybrid by increasing specific surface area and promoting disparity of Co NPs, which synergistically enhanced catalytic activity [14].

**Fig. 3:**
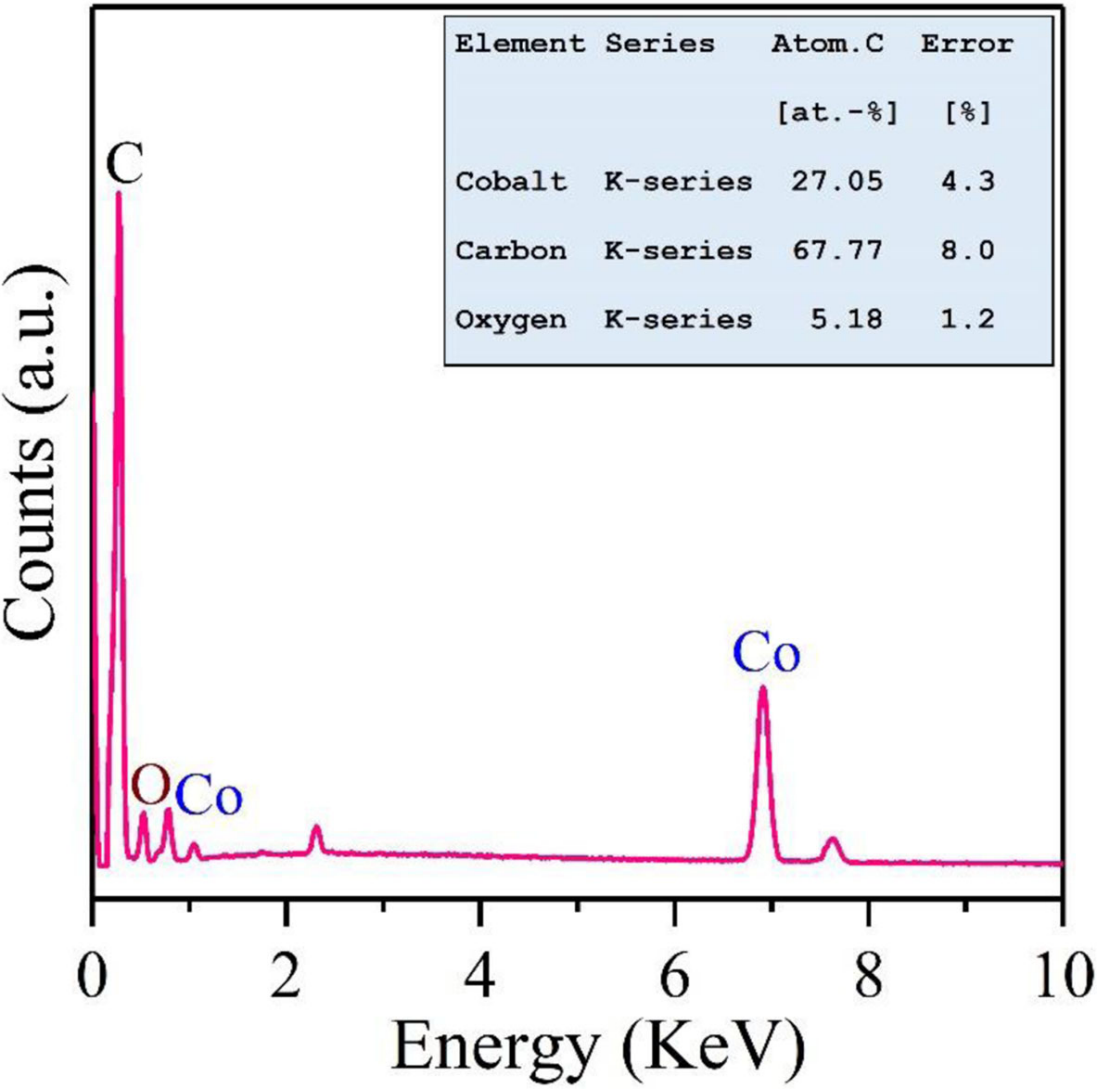
EDX spectrum of Co-rGO nanohybrid. The inset image shows obtained elemental mapping results of Co-rGO sample

Energy-dispersive X-ray (EDX) analysis was used to investigate information of localized elements inside Co-rGO nanocomposite. Figure 3 shows the elemental analysis of Co-rGO nanohybrid, which clearly displays the existence of C, O, and Co elements inside the sample. The inset image of Fig. 3 demonstrates the atomic percentage (at %) of constituent elements in Co-rGO nanohybrid. The atomic percentages of Co, C, and O were found to be 27.05, 67.77, and 5.18, respectively in the nanohybrid.

**Fig. 4:**
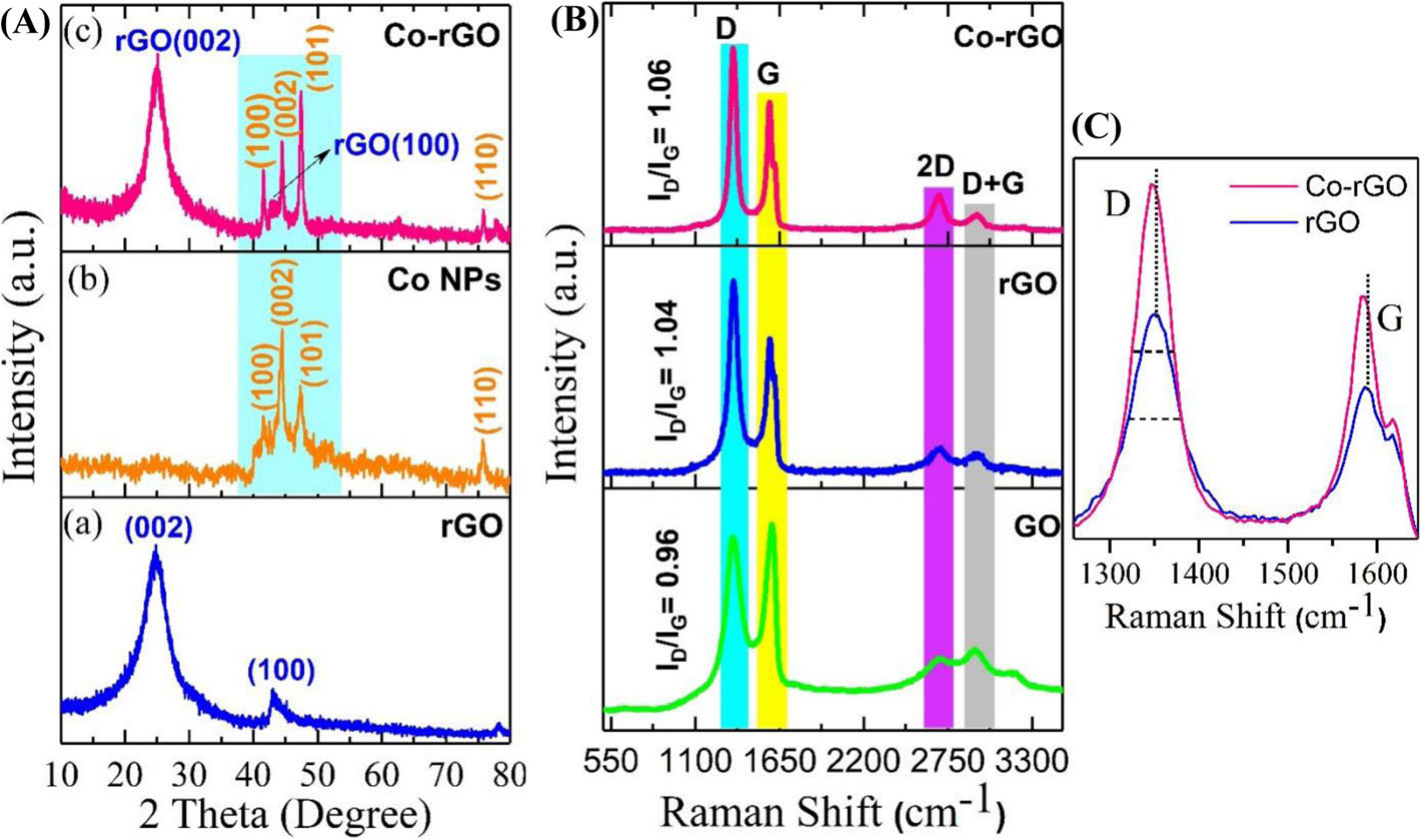
(**A**) XRD patterns **a** rGO nanosheets, **b** Co NPs, and **c** Co-rGO nanohybrid (B) Raman spectra of GO, rGO, and Co-rGO nanohybrid (bottom to top). (C) Raman spectra of rGO and Co-rGO, showing changes in the D and G peaks

Figure 4a shows the XRD pattern of the rGO nanosheets. It describes the successful exfoliation of GO into rGO as it contains reflection from (002) and (100) planes at 24.83° and 43°, respectively [15]. XRD pattern of Co NPs successfully indexed with (100), (002), (101), and (110) planes at 41.63°, 44.24°, 47.37°, and 75.80°, respectively of Co (Fig. 4Ab) [14]. These reflection planes are well consistent with the hcp structure of Co NPs (JCPDS No. 05-0727). This exciting phase was observed due to the conversion of cobalt acetylacetonate [Co(acac)_3_] into metallic cobalt by the solvothermal reaction. Further, Fig. 4Ac show the XRD pattern of Co-rGO nanohybrid. In addition to reflection planes observed in rGO {(002), (100) at 24.83° and 43°, respectively}, the XRD pattern contains all reflection planes as found in the case of Co NPs. This result indicates a proper phase formation of Co NPs on the surface of rGO nanosheets. It is also noticed from Fig. 4Ac that the relative intensity of Co peaks increased with the formation of Co-rGO nanohybrid. This could be attributed to enhancement in the crystallinity and orientation of Co NPs due to rGO. Xu et al. observed similar characteristics in the case of Co-rGO nanocomposite [14].

Figure 4b shows the Raman spectra of GO, rGO, and Co-rGO nanohybrid. All three materials contain four bands, namely D, G, 2D, and D + G, with a slight change in their wave number. The G-band of rGO and Co-rGO nanohybrid appears at 1586 cm^−1^ and 1585 cm^−1^ respectively, whereas G-band of GO is observed at 1600 cm^−1^ [15, 16]. Compared with GO, the G-band of rGO and Co-rGO gets shifted towards lower wavenumber, indicating the reduction of GO into rGO [15]. In general, the origin of D band is considered to be disorders of carbon atoms as well as a defect in the graphitic structure, whereas G-band is referred to sp^2^ hybridization of ordered carbon atoms in E_2g_ vibration mode [18, 19]. Further, the intensity ratio of the D and G band (I_D_/I_G_) roughly calculates the defect extent and degree of graphitization of carbon atoms. The I_D_/I_G_ values for rGO and Co-rGO were found to be 1.04 and 1.06, respectively, which are higher than the I_D_/I_G_ value of GO (0.96). Moreover, it is observed from Fig. 4c that the D and G bands of hybrid shifted towards lower wavenumber (redshift), and its full-width half maxima are also changed in comparison to rGO. These results indicate the hybridization of rGO and Co orbitals and confirm strong electronic interaction between rGO and Co in the hybrid structure [8, 20] The splitting in G-band of both rGO and Co-rGO hybrid (Fig. 4c) established that the rGO sheets are not more than trilayer-graphene [21].

**Fig. 5:**
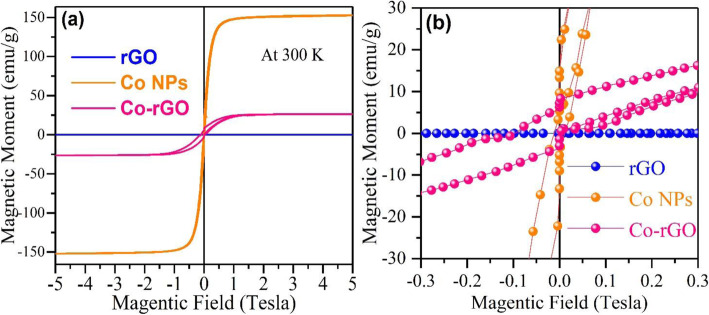
**a** Room temperature M-H graph of rGO, Co NPs, and Co-rGO nanohybrid. **b** M-H graph in the lower field region (− 0.3 T to + 0.3 T).

**Fig. 6:**
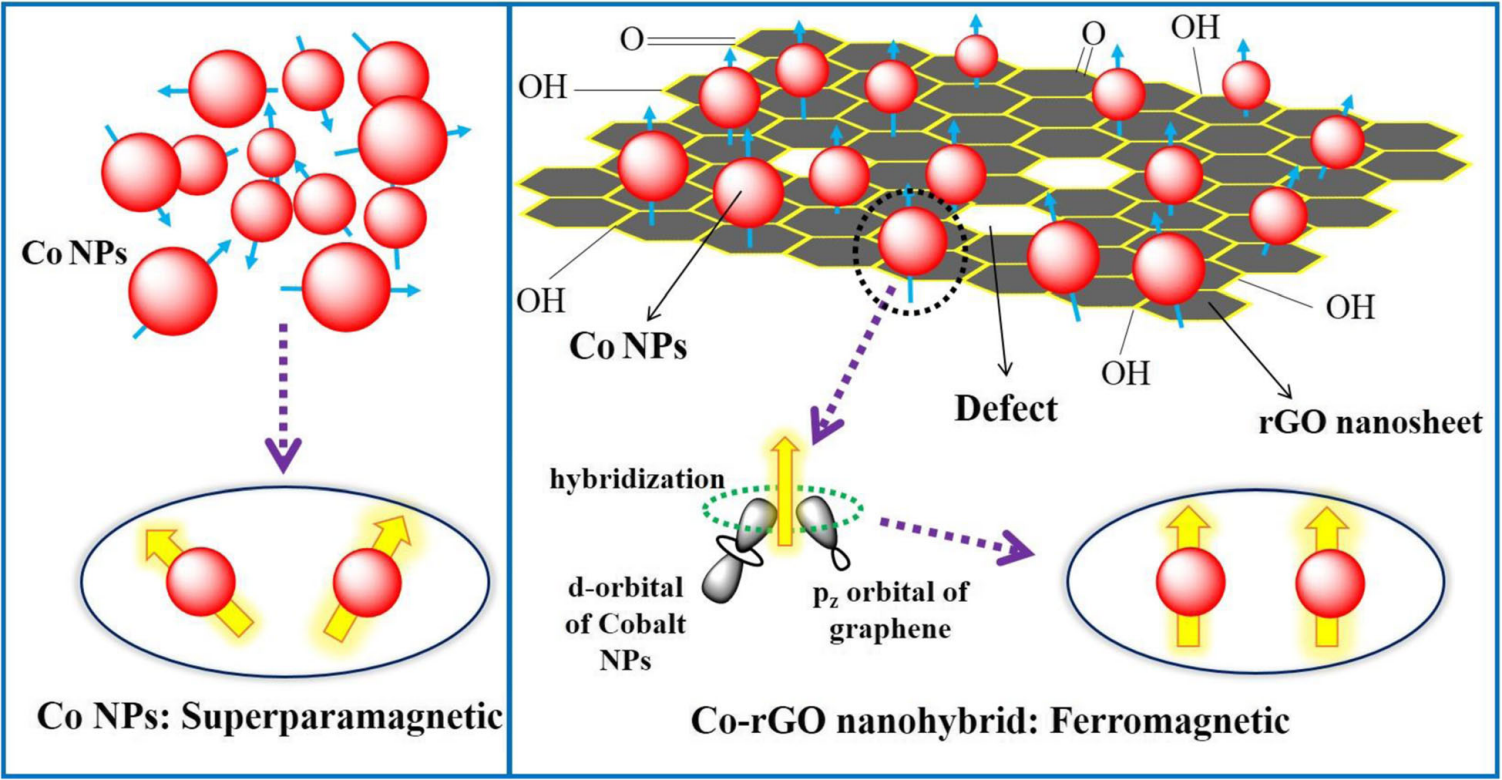
Schematic model for SPM and FM behavior in Co NPs and Co-rGO nanohybrid, respectively

Figure 5a demonstrates the room temperature field-dependent magnetization (M-H) curve for rGO, Co NPs, and Co-rGO nanohybrid. It reveals that the rGO nanosheet has a non-magnetic response, as expected. On the other hand, Co NPs depict superparamagnetic behavior (minimal coercivity, H_C_ 115 Oe) [22, 23]. This behavior observed due to the smaller size of Co NPs (below 20 nm) [24]. In this condition, the thermal energy becomes comparable with anisotropic magnetic energy and leads to the flipping of spins in a short period of time (Fig. 6 left panel). The magnetic anisotropy energy *E* (*ϴ*) per particle is defined as “the energy required to hold the magnetic moment in a particular direction” and can be expressed as

*E* (*ϴ*) = *K*_*eff*_*V* Sin^2^*ϴ*

Here, *K*_eff_ is anisotropy constant, *V* is the particle volume, and *ϴ* is the angle between magnetization and easy axis [22]. The magnetic anisotropic energy barrier that separates the two spins, i.e., spin-up and spin-down state, is proportional to *K*_*eff*_*V*. When the size of the NC decreases sufficient enough, the energy barrier becomes smaller than the thermal energy (K_B_T), resulting in the flipping of spins. This behavior is called superparamagnetic and material is called super-paramagnet. Such material has a massive magnetic moment in a small magnetic field with no hysteresis at all. From the M-H loop, the calculated value of coercivity (H_C_), remanent magnetization (M_R_), and saturation magnetization (M_S_) for Co NPs and Co-rGO nanohybrid are listed in Table 1.

**Table 1:** Observed results from the M-H curve of Co NPs and Co-rGO nanohybrid

Samples	Saturation magnetization (M_**S**_) emu/g	Remanent Magnetization (M_**R**_) emu/g	Coercivity (H_**C**_) Oe
**Co NPs**	150	3.50	115
**Co-rGO nanohybrid**	26	5.90	650

Further, the M-H curve of CO-rGO nanohybrid, as shown in Fig. 5, illustrates ferromagnetic behavior since its magnetization almost saturated with high H_C_ and M_R_ values 650 Oe and 5.90 emu/g, respectively. Nevertheless, the M_S_ value of Co-rGO nanohybrid significantly reduced to 26 emu/g in comparison to its bulk M_S_ value (168 emu/g) [24]. It is because of non-magnetic rGO nanosheets and smaller size of Co NPs. The origin of ferromagnetism (FM) in Co-rGO nanohybrid is believed to result from the hybridization of p_z_-orbital of rGO with d-orbital of Co NPs (Fig. 6 right panel). This results in partial electron transfer from rGO to Co d-orbital, which further modified the electronic states of nanohybrid and promoting the ferromagnetic interaction. As discussed earlier, the Raman spectrum of hybrid depicts a strong electronic interaction between Co and rGO, confirmed that charge transfer between Co and rGO. Sun et al*.* observed similar characteristics in the case of rGO rapped Co-doped ZnO (Co: ZnO) quantum dots [8]. They have proposed that rGO can form Co^2+^-V_O_ complex in the Co: ZnO because of charge transfer from rGO to Co: ZnO. This leads to changes in the fermi level and results in the observation of room temperature ferromagnetism in the hybrid structure.

Thus, with the formation of Co-rGO hybrid, the superparamagnetic interaction between Co NPs transformed into long-range ferromagnetic interaction. The other microstructural parameters, such as morphology, internal stress, and orientation defects, can also enhance the coercivity value [25, 26]. The magnetization results from present work have been compared with some MNPs-rGO nanocomposite and listed in Table 2.

**Table 2:** Comparison of magnetic properties of present work with reported MNPs-rGO composite

Nanocomposite	H_C_ (Oe)	M_S_ (emu/g)	M_R_ (emu/g)	Magnetic behavior	Reference
Fe_3_O_4_/rGO	……	10.23	0.03	Superparamagnetic	[27]
CoFe_2_O_4_/rGO	410	75.37	20.05	Superparamagnetic	[20]
Ni/rGO	179	19	8	Ferromagnetic	[28]
Co/rGO	226	53.4	6	Ferromagnetic	[26]
Co-rGO	650	26	5.90	Ferromagnetic	Present work

## Conclusions

We have used a one-step solvothermal method to prepare cobalt nanoparticles and reduced graphene oxide and its derivative forming Co-rGO nanohybrid. XRD, TEM, SEM, and EDX characterization techniques were used to confirm the proper formation of Co-rGO nanohybrid. Significant changes in the Raman spectrum of Co-rGO nanohybrid indicates intensive electronic interaction between rGO and Co in the nanohybrid. The observation of room-temperature ferromagnetism in the Co-rGO nanohybrid could be a result of electronic interaction between rGO and Co NPs, which further promotes magnetic interaction through long-range ordering. Thus, this study opens up a possibility to synthesize ferromagnetic Co-rGO nanohybrid, which could be beneficial for future spintronics, catalysis, and MRI applications.

## Data Availability

The used datasheets and materials are available from corresponding author on reasonable request.

## Abbreviations

NPs: Nanoparticles
MNPs: Magnetic nanoparticles
Co: Cobalt
Fe: Iron
Ni: Nickel
GO: Graphite oxide
rGO: Reduced graphene oxide
MRI: Magnetic resonance imaging
2D: Two dimensional
M-H: Field-dependent magnetization
XRD: X-ray diffractometer
SEM: Scanning electron microscope
EDX: Energy-dispersive X-ray
TEM: Transmission electron microscope
HRTEM: High-resolution TEM
VSM: Vibrating sample magnetometer
at %: Atomic percentage
SPM: Superparamagnetism
FM: Ferromagnetism
Oe: Oersted
T: Tesla
M_S_: Saturation magnetization
M_R_: Remanent magnetization
H_C_: Coercivity
